# Spontaneous Shape Alteration and Size Separation of Surfactant-Free Silver Particles Synthesized by Laser Ablation in Acetone during Long-Period Storage

**DOI:** 10.3390/nano8070529

**Published:** 2018-07-13

**Authors:** Dongshi Zhang, Wonsuk Choi, Jurij Jakobi, Mark-Robert Kalus, Stephan Barcikowski, Sung-Hak Cho, Koji Sugioka

**Affiliations:** 1 RIKEN Center for Advanced Photonics, 2-1 Hirosawa, Wako, Saitama 351-0198, Japan; dongshi17@126.com (D.Z.); cws@kimm.re.kr (W.C.); 2 Department of Nano-Mechatronics, Korea University of Science and Technology (UST), 217 Gajeong-Ro, Yuseong-Gu, Daejeon 34113, Korea; shcho@kimm.re.kr; 3 Department of Nano-Manufacturing Technology, Korea Institute of Machinery and Material (KIMM), 156 Gajeongbuk-Ro, Yuseong-Gu, Daejeon 34103, Korea; 4 Technical Chemistry I and Center for Nanointegration Duisburg-Essen (CENIDE), University of Duisburg-Essen, Universitaetsstrasse 7, 45141 Essen, Germany; jurij.jakobi@uni-due.de (J.J.); mark-robert.kalus@uni-due.de (M.-R.K.); stephan.barcikowski@uni-due.de (S.B.); 5 Department of Laser & Electron Beam Application, Korea Institute of Machinery and Material (KIMM), 156 Gajeongbuk-Ro, Yuseong-Gu, Daejeon 34103, Korea

**Keywords:** laser ablation in liquids, particle growth, carbon encapsulation, polygonal particle, core-shell particle, surfactant-free

## Abstract

The technique of laser ablation in liquids (LAL) has already demonstrated its flexibility and capability for the synthesis of a large variety of surfactant-free nanomaterials with a high purity. However, high purity can cause trouble for nanomaterial synthesis, because active high-purity particles can spontaneously grow into different nanocrystals, which makes it difficult to accurately tailor the size and shape of the synthesized nanomaterials. Therefore, a series of questions arise with regards to whether particle growth occurs during colloid storage, how large the particle size increases to, and into which shape the particles evolve. To obtain answers to these questions, here, Ag particles that are synthesized by femtosecond (fs) laser ablation of Ag in acetone are used as precursors to witness the spontaneous growth behavior of the LAL-generated surfactant-free Ag dots (2–10 nm) into different polygonal particles (5–50 nm), and the spontaneous size separation phenomenon by the carbon-encapsulation induced precipitation of large particles, after six months of colloid storage. The colloids obtained by LAL at a higher power (600 mW) possess a greater ability and higher efficiency to yield colloids with sizes of <40 nm than the colloids obtained at lower power (300 mW), because of the generation of a larger amount of carbon ‘captors’ by the decomposition of acetone and the stronger particle fragmentation. Both the size increase and the shape alteration lead to a redshift of the surface plasmon resonance (SPR) band of the Ag colloid from 404 nm to 414 nm, after storage. The Fourier transform infrared spectroscopy (FTIR) analysis shows that the Ag particles are conjugated with COO– and OH– groups, both of which may lead to the growth of polygonal particles. The CO and CO_2_ molecules are adsorbed on the particle surfaces to form Ag(CO)_x_ and Ag(CO_2_)_x_ complexes. Complementary nanosecond LAL experiments confirmed that the particle growth was inherent to LAL in acetone, and independent of pulse duration, although some differences in the final particle sizes were observed. The nanosecond-LAL yields monomodal colloids, whereas the size-separated, initially bimodal colloids from the fs-LAL provide a higher fraction of very small particles that are <5 nm. The spontaneous growth of the LAL-generated metallic particles presented in this work should arouse the special attention of academia, especially regarding the detailed discussion on how long the colloids can be preserved for particle characterization and applications, without causing a mismatch between the colloid properties and their performance. The spontaneous size separation phenomenon may help researchers to realize a more reproducible synthesis for small metallic colloids, without concern for the generation of large particles.

## 1. Introduction

Laser ablation in liquids (LAL) is increasingly gaining worldwide attention as a newly emerging bottom-up and top-down combined technique for novel nanomaterial synthesis, offering diverse applications including optics and biology [1,2,3,4]. In particular, the LAL-synthesized nanomaterials possess a higher purity than the counterparts synthesized by wet-chemistry techniques [1], because surfactants or stabilizers are not required for colloid synthesis, which makes them very attractive and competitive for catalytic applications [5,6,7,8]. High-purity nanomaterial means that a large amount of active sites are exposed to the surrounding reactants or nanoparticles (NPs), which is actually a double-edged sword in nanoscience [7]. This is because spontaneous particle growth [9,10,11,12] may occur, which can increase the difficulty in accurately controlling the sizes [13,14,15,16] and the shapes [17,18,19,20,21] of the as-prepared nanomaterials.

Six mechanisms have been proposed for the growth of the LAL-generated nanomaterials, including LaMer-like growth [9], coalescence [22], Ostwald ripening [10], particle (oriented) attachment [18,23,24,25], adsorbate-induced growth [18,26], and reaction-induced growth [17]. Unlike the wet-chemistry synthesis method, where particle growth terminates when the seed concentration drops below the critical concentration [27], the particle growth can be sustained during the entire period of LAL, because every pulse ablation produces new seeds for particle growth. This is the reason novel football-like AgGe microspheres [17], with sizes up to 7 μm, can be obtained by LAL. Another difference from the conventional wet-chemistry techniques is the possibility of inducing multiple growth processes to generate different nanostructures. For example, large (>100 nm) hollow Mn_3_O_4_ spheres are formed from ca. 20 nm cubic particles that are assembled from smaller 5–8 nm smaller particles after a long-period LAL [18]. Previously, the investigation on particle growth has mainly been on the fragmentation of large metallic particles, such as Pt [10] and Au [11] into 2~3 nm particles, and then their evolution over time is observed. No attempt has been made to study the growth of the metallic particles just after laser irradiation, which occupies the dominant position in all three liquid-phase laser synthesis methodologies, LAL, laser fragmentation in liquids (LFL), and laser melting in liquids (LML) [1].

LAL is currently most frequently implemented in a batch chamber where the ablation is often accompanied by LFL when the particles enter the beam path in the liquids [1]. The fragmentation behavior of the particles may become dominant when implementing the LAL for a long period. Long-period LAL means that the LAL should last more than half an hour, in order to generate highly saturated colloids in a limited volume of liquids [9]. In this case, a large amount of active surfactant-free particles are generated by LFL, which increases the chance for the particles to encounter each other by Brownian motion, and then to grow overtime during storage, transportation, and when employed in applications. If growth does occur, then it should attract special attention from academia, because until now, the characterization and application of metallic particles such as Ag NPs [28,29,30,31] have been seldom described in the literature. Therefore, it is not clear whether the size information shown in the literature has been obtained from either freshly prepared colloids or after a certain period of growth. The particle growth of the metallic particles is also accompanied by the shape transformation into nonspherical nanostructures, such as nanowires [10,11]. Tsuji’s group reported that the post laser irradiation of LAL-generated Ag colloids in either a citrate [32], polyvinylpyrrolidone (PVP) aqueous solution [33], water [34], or acetone-water mixed solution [35] may cause the formation of nanoplates and nanorods during colloidal aging [36]. However, it is still unknown whether the Ag particles synthesized by long-period LAL can still grow into polygonal products, and the maximal size at which the particle growth terminates is also unknown, especially in the case of LAL in organic solvents, where the solvent decomposition definitely occurs to generate a large amount of carbon or hydrocarbons, which may precipitate on the active particles to terminate the particle growth.

In this paper, one hour long-period LAL of Ag in acetone, using a femtosecond laser (fs-LAL), was implemented to investigate the particle growth behavior. Laser powers of 300 mW and 600 mW were used to change the productivity of the Ag colloids, as well as the amount of both ultra-small particles and large particles with sizes less than and greater than 10 nm, respectively. The variation in the surface plasmon resonance (SPR) band of the Ag colloids during storage was recorded using UV-VIS spectroscopy, while the particles’ sizes before and after the six month storage were analyzed using transmission electron microscopy (TEM), both of which provide evidence for particle growth and newly discovered size separation phenomena. The surface chemistry of the Ag NPs was analyzed using Fourier transform infrared spectroscopy (FTIR), to identify the chemical groups that may be responsible for the particle growth. This study was complemented by nanosecond (ns) laser ablation to demonstrate that particle growth is not limited to fs-LAL. Finally, the scenarios for both the particle growth and the size separation phenomena are proposed.

## 2. Results and Discussion

### 2.1. Femtosecond Laser Ablation

**Figure 1:**
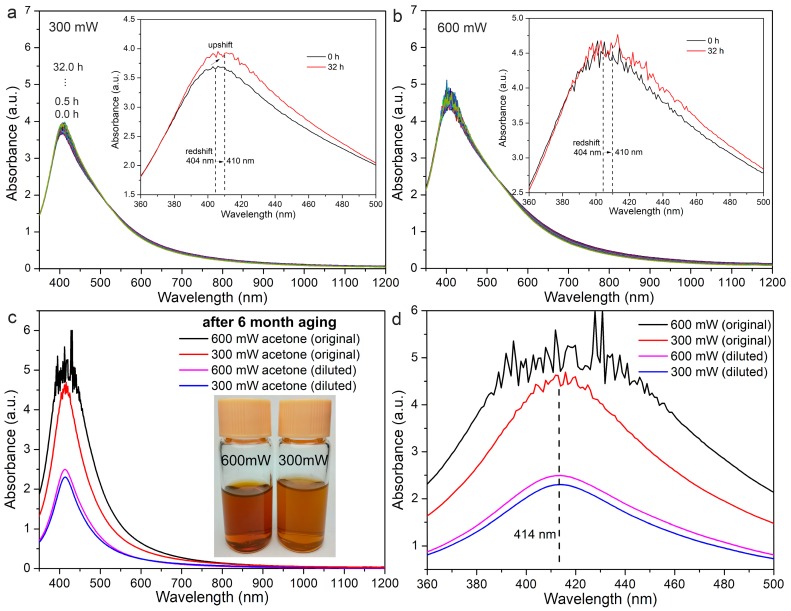
Absorption spectra for Ag nanoparticles (NPs) synthesized by laser ablation of Ag in acetone, and then stored for various periods. Upper: spectra for samples prepared at a laser power of (**a**) 300 mW and (**b**) 600 mW, and stored within 32 h (insets: enlarged spectra in the wavelength range of 360 nm to 500 nm for samples stored for 0 and 32 h). Lower: spectra for (**c**) samples prepared at laser powers of 300 mW and 600 mW, and stored for six months (inset: photos of aged Ag NPs in acetone), and (**d**) enlarged spectra in the wavelength range of 360 nm to 500 nm (black and red curves: as-aged colloidal samples; pink and blue curves: diluted as-aged samples prepared by the addition of pure acetone to the colloids).

It has been reported that the redshift of the SPR peaks for the Ag NPs is often indicative of an increase of the particle size [37] via either particle ripening or coalescence, thus giving indirect evidence for particle growth. In our experiments, the variations in the absorption spectra of the fresh Ag colloids synthesized by LAL at laser powers of 300 mW and 600 mW, were recorded within 32 h, with a time interval of 30 min, as shown in Figure 1a,b. As the aging time increases, in both cases, the colloidal SPR peaks redshift from 404 nm to 410 nm, which suggests the spontaneous particle growth during colloidal storage. The higher SPR peak intensity of Ag colloids synthesized at 600 mW compared with those synthesized at 300 mW, indicates that the LAL at a higher laser power gives rise to a slightly higher productivity of the colloids. A slight increase in the SPR peak intensity with aging is observed in both cases, which is attributed to the increased concentration of the Ag NPs, caused by the evaporation of acetone. After six months of storage, during which time the growth was already terminated, the absorption spectra of the colloids were characterized again, as shown in Figure 1c,d. It is clear that the evaporation of the acetone further increases the colloidal concentrations, even though the colloids were sealed with acetone in glass containers. To compensate for the evaporated acetone, the colloids were diluted with additional acetone, and characterized (pink and blue curves in Figure 1c). The magnified SPR peaks in the wavelength range of 360–500 nm show a further redshift to 414 nm in both cases, which indicates that particle growth should continue even after the 32 h of storage. The inset figures in Figure 1c show photographs of the colloids in acetone after six months of storage. Both of the Ag colloids are orange colored. The higher concentration of Ag colloids prepared at a higher power is evident from the darker orange color. These colors are different from the light yellow color of the Ag colloids reported previously [38], probably due to the much higher concentration of Ag colloids presented in this work. Particle precipitation was also observed at the bottom of the glass containers. Therefore, a subsequent analysis of both of the stable colloids in liquids, and the particles precipitated at the bottom of the glass containers, was performed.

**Figure 2:**
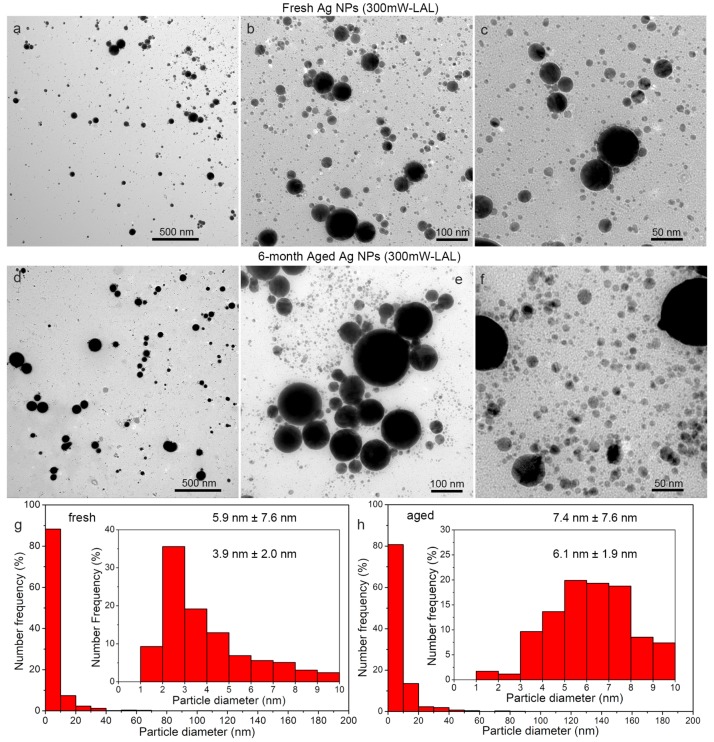
TEM images (**a**–**f**) and size distributions (**g**,**h**) of fresh (**a**–**c**,**g**) and six month (**d**–**f**,**h**) aged Ag particles, synthesized by laser ablation of Ag in acetone, at a laser power of 300 mW.

**Figure 3:**
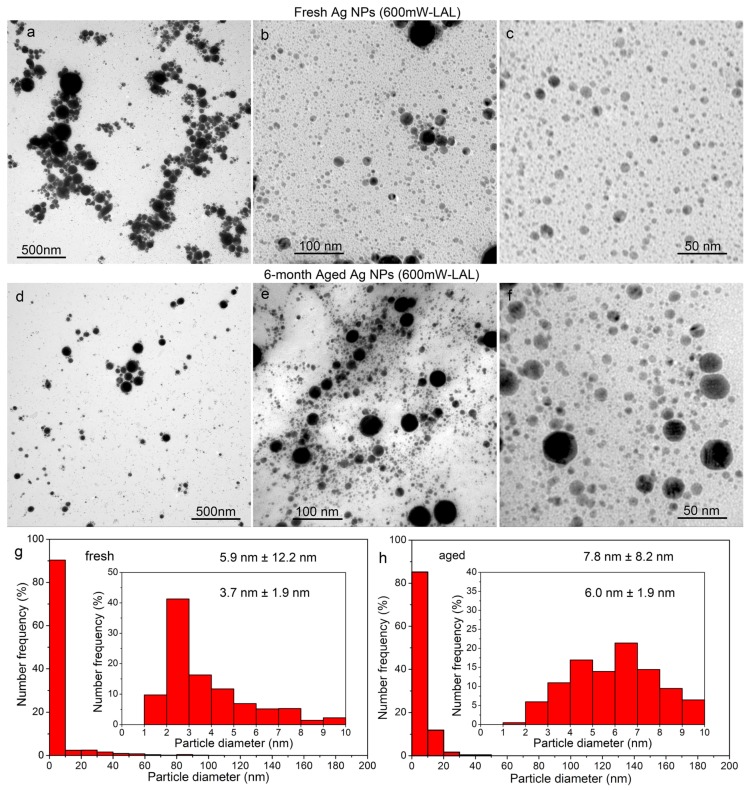
TEM images (**a**–**f**) and sizes distributions (**g**,**h**) of fresh (**a**–**c**,**g**) and six month (**d**–**f**,**h**) aged Ag particles, synthesized by laser ablation of Ag in acetone at laser power of 600 mW.

Figure 2 and Figure 3 show the TEM images and the calculated size distributions of the fresh and six month aged Ag particles generated by LAL, at the laser powers of 300 mW and 600 mW, respectively. In accordance with the redshift of the SPR peaks, shown in Figure 1a,b, particle growth indeed occurs (Figure 2a–f and Figure 3a–f), especially for ultrasmall particles with sizes less than 10 nm. The average sizes of the fresh Ag particles synthesized at 300 mW and 600 mW were evaluated to be 5.9 ± 7.6 nm and 5.9 ± 12.2 nm (Figure 3g), respectively, which confirmed that the average particle size was almost independent of the laser power. However, a greater amount of particles larger than 10 nm were generated at a higher laser power of 600 mW (Figure 2a vs. Figure 3a), as indicated by the increased ratio between the big and small particles (Figure 2g vs. Figure 2h). The majority of the Ag NPs are in the range of 1–10 nm, occupying ca. 90% of the total amount for both of the cases. A further subdivision of the size distributions of the Ag NPs shows that most of the particles are ca. 2 nm (Figure 2c and Figure 3c). After six months of storage, the average sizes of the Ag colloids increase to 7.4 ± 7.6 nm and 7.8 ± 8.2 nm, respectively. In the case of the colloids synthesized at 600 mW, a significant decrease in the large particles was observed (Figure 3a,d).

In both cases, the number ratios of the as-aged colloids with sizes in the range of 10–20 nm increased to 12–14% after 6 months of storage. The number ratios of the as-aged colloids with a diameter of 20~30 nm were almost the same as those of the fresh colloids (Figure 2g,h and Figure 3g,h). The number ratio of the as-aged colloid with a diameter of 30~40 nm increased slightly for the as-aged 300 mW LAL colloid, but decreased for the as-aged 600 mW LAL colloid. The number ratios of the Ag NPs with diameters larger than 40 nm were less than 1%, which is almost negligible for both cases, so it is difficult to quantify the variation in the number ratios of the colloids. However, a comparison of the particle morphologies of the fresh and as-aged colloids clearly shows that the number ratio of the particles larger than 40 nm did not change significantly (Figure 2a vs. Figure 2d) after six months of storage for the 300 mW LAL colloid, but did decrease significantly for the as-aged 600 mW LAL colloid (Figure 3a vs. Figure 3d).

**Figure 4:**
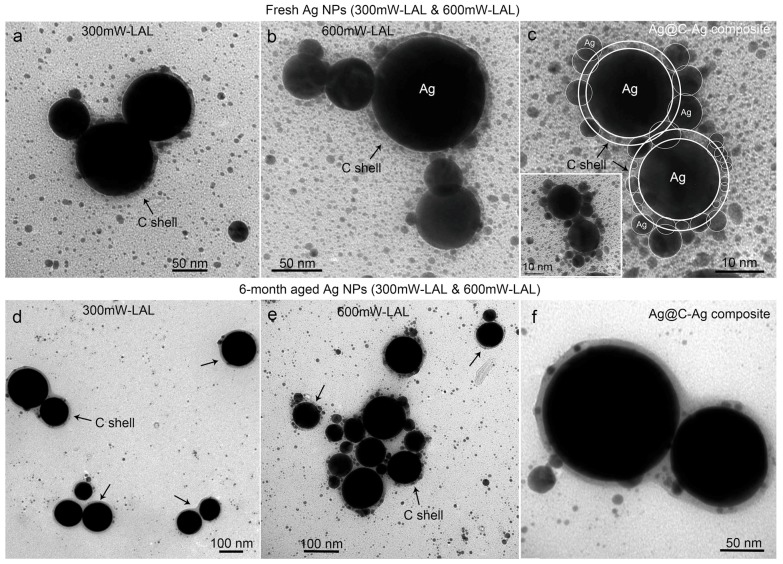
TEM images of the Ag@C core-shell particles observed in (**a**–**c**) fresh and (**d**–**f**) six month aged colloids synthesized by laser ablation in liquids (LAL), at laser powers of 300 mW (**a**,**d**) and 600 mW (**b**,**e**). The TEM images in (**c**,**f**) show the Ag@C-Ag composites from the fresh and the six month aged colloids.

**Figure 5:**
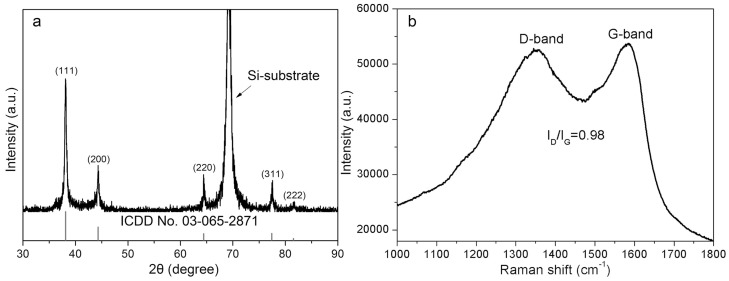
(**a**) XRD pattern and (**b**) Raman spectrum of the LAL-synthesized Ag NPs.

In more detailed observations of large particles, regardless of them being fresh (Figure 4a–c) or six months aged colloids (Figure 4d–f) prepared at laser powers of 300 mW and 600 mW, the carbon-encapsulated particles with core sizes ranging from 25 nm to ca. 200 nm, and a carbon shell thickness of 3~10 nm are all found, as shown in Figure 4. Some smaller particles were captured by the carbon shells, which led to the formation of Ag@C-Ag truffle-like aggregates. The crystalline structure of the Ag cores was confirmed by X-ray diffraction (XRD) characterization, as shown in Figure 5a. The featured peaks of the colloids fit well with the standard card for Ag (ICCD No. 03-065-2871). The presence of the carbon shells was verified by Raman spectroscopy observation of both the D-band (1360 cm^−1^) and G-band (1582 cm^−1^) peaks of carbon (Figure 5b). The ratio of the D-band to the G-band peak intensities was calculated to be 0.98. The G-band is associated with the ordered graphite (sp^2^) structure, while the D-band is related to the disordered graphite layers, such as soot, chars, glassy carbon, and evaporated amorphous carbon [39]. Consequently, it can be concluded that the carbon shells possess high ratios of disorders. According to Robertson [39], the carbon shells that are composed of both crystalline graphite and amorphous carbon can be assigned to diamond-like carbon (DLC), a metastable form of carbon. Because of the presence of abundant hydrogen, which are generated from the LAL-induced acetone decomposition, it is highly possible that other products, such as hydrogenated amorphous carbon (a-C: H) and tetrahedral amorphous carbon (ta-C) [39] also form during LAL. Nevertheless, their existence cannot be confirmed by the Raman spectrum, shown in Figure 5b. The identification and quantification of different carbon disorders will be a focus of our future studies.

**Figure 6:**
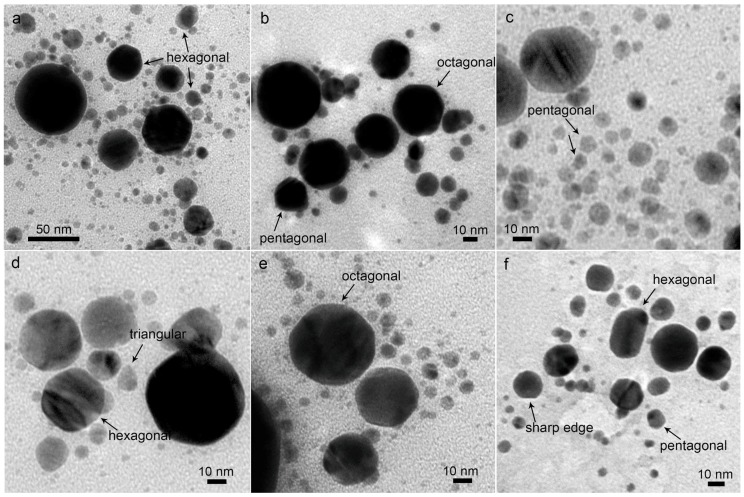
(**a**–**f**) Polygonal particles obtained after six months of storage of the colloids.

Some polygonal Ag nanocrystals, such as triangular (Figure 6d), pentagonal (Figure 6c,f), hexagonal (Figure 6a,d,f), octagonal (Figure 6b,e), and some spherical particles with sharp edges, (Figure 6f) were observed, of which the sizes were in the range of 5–50 nm, similar to the Ag nanocrystals prepared by the reduction of AgNO_3_ [40]. The maximal sizes of the newly grown Ag nanocrystals are much smaller than the 100~500 nm crystals obtained by the post-irradiation of the LAL-generated Ag spheres [33], which indicates that the surrounding carbon atoms may cover the newly formed nanocrystals and inhibit their further growth into larger particles. The following results provide evidence for the spontaneous growth of small particles into nonspherical Ag nanocrystals: (1) the amount of the ultrasmall particles, of 2–3 nm in diameter, is significantly decreased after storage (Figure 2g,h and Figure 3g,h); (2) polygonal particles are not surrounded by ultrasmall particles (Figure 6), unlike the spherical Ag NPs encapsulated by carbon shells (Figure 4); and (3) previous reports show that the size of the Ag NPs that correspond to an SPR band of 414 nm is around 30 nm [41], which is much larger than the ca. 8 nm (Figure 2h and Figure 3h) of the six month aged Ag colloids with the same SPR position (Figure 1c,d) in this work. Considering that the polygonal Ag nanostructures, such as the hexagonal and triangular Ag nanoplates, often have a higher SPR band position than the Ag spheres with the same sizes [42], it is reasonable to deduce that the formation of the polygonal particles is the main contribution to the redshift of the SPR band during the storage of the colloids, rather than a change of the particle size. From the occurrence of particle growth, the building blocks (fresh ultrasmall Ag NPs) should be surfactant-free, which facilitates the attachment and ripening of ultrasmall particles into various polygonal nanoparticles, with the growth direction governed by the surface bindings [9,18], which will be discussed below.

**Figure 7:**
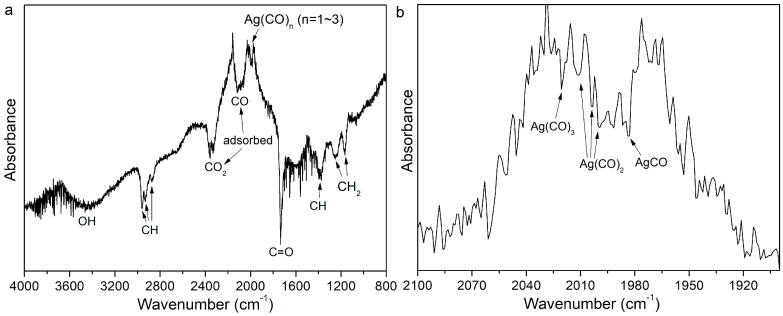
(**a**) FTIR spectrum of the six month aged Ag NPs and the (**b**) magnified spectrum where the peaks of Ag(CO)_n_ (n = 1–3) complexes are located.

To determine the surface groups that may direct the particle growth into the polygonal particles, the six month aged Ag particles were analyzed using FTIR, and the results are shown in Figure 7. The vibration bands at 1165 cm^−1^ and 1250 cm^−1^ are assigned to the wagging and the rocking vibrations of CH_2_, respectively, while the peaks at 1375 cm^−1^ and 1734 cm^−1^ are assigned to the stretching vibrations of C–H alkane [43] and C=O [44], respectively. The broad peak between 3000 cm^−1^ and 3500 cm^−1^ is because of the OH stretching [44]. The broad bands of 2061~2158 cm^−1^ and 2325~2343 cm^−1^ are related to the stretching vibrations of the adsorbed CO [45,46] and CO_2_ [47] molecules, respectively. Therefore, the CO-metal [48] and CO_2_-metal complexes [49], such as Ag(CO)_n_ (n = 1~3) [50] and Ag–O–C–O, may be generated during LAL (Figure 7b). The three vibration peaks at 2902 cm^−1^, 2932 cm^−1^, and 2960 cm^−1^, are due to the CH stretching from the alkyl groups [44]. The FTIR analysis indicates that the acetone molecules are decomposed into CO_2_, CO, alkanes, and OH– groups during LAL, which may either strongly adsorb onto the Ag NPs or interact with the Ag NPs to form Ag(CO)_n_ or Ag(CO_2_)_n_ complexes. As a result, the colloids are endowed with ultrahigh stability, which enables them to self-stabilize, even after six months of storage. Tsuji’s group confirmed that shape transformation from the Ag spheres into prisms is not induced by acetone molecules, but should be caused by water molecules. In the present case, considering the absence of water molecules in the liquids, the OH– formed by the acetone decomposition and reconstruction is one possible candidate to cause the anisotropic growth of Ag particles [35] via the Ostwald ripening process [33]. Chemical adsorbed carboxylate (COO–) groups may be another candidate to trigger the selective Ag crystal facet growth during ripening [51].

**Figure 8:**
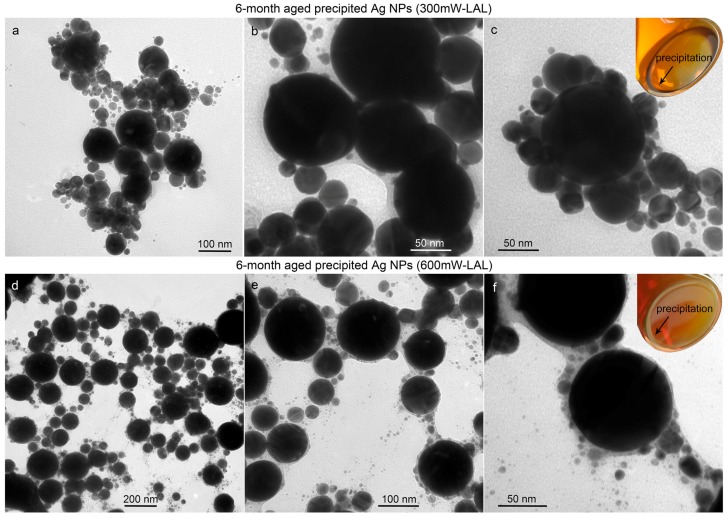
Precipitated particles after the six month storage of the Ag colloids, synthesized by femtosecond LAL at laser powers of (**a**–**c**) 300 mW and (**d**–**f**) 600 mW, respectively. The arrows shown in the optical images in the insets (**c**,**f**) indicate the particle precipitation after six months storage.

**Figure 9:**
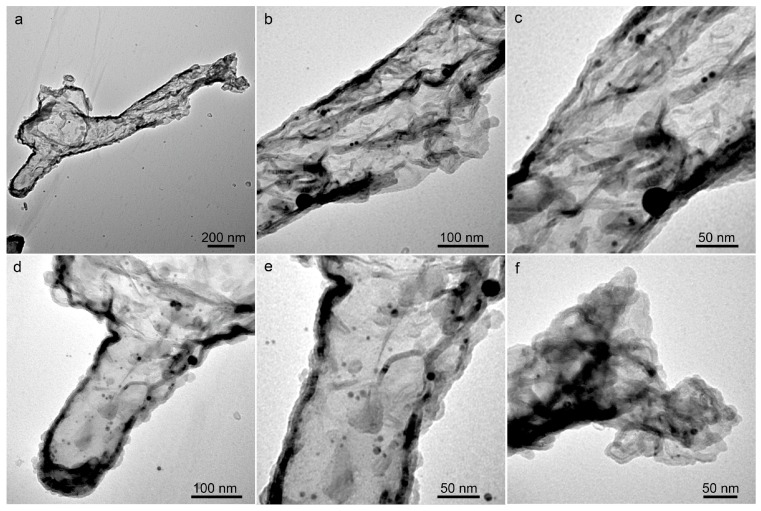
(**a**–**f**) TEM images of the carbon nanosheet formed during storage of the colloids.

The optical images of the six month aged colloids show that some particles have already precipitated during storage (inset images in Figure 8c,f). In comparison with the particle morphologies of the fresh and six month aged colloids synthesized by LAL at a laser power of 600 mW (Figure 3a,d), it is also clear that the amount of particles with diameters larger than 40 nm decreased dramatically after a long-period storage (Figure 3g,h), which is due to the precipitation of larger particles. Figure 8 shows the TEM images of the precipitated particles prepared at laser powers of 300 mW and 600 mW after six months of storage. The precipitated particles are mainly particles larger than 40 nm, which are encapsulated by carbon to form particle networks, which also encapsulate a certain amount of particles with sizes less than 40 nm, so that the number ratios of the ultrasmall colloids (1–10 nm) decrease (Figure 2h and Figure 3h). Despite the precipitation induced by the carbon capture, the number ratios of the colloids with sizes of 10–20 nm still increase, and the number ratios of the colloids with sizes of 20–30 nm remain almost unchanged. This means that the continuous supplement of the Ag NPs with sizes of 10–30 nm as a result of the particle growth of the ultrasmall surfactant-free Ag NPs occurs. An analysis of the change in the colloidal size distribution after six months of storage (Figure 2g,h and Figure 3g,h) led us to conclude that the colloids synthesized by LAL at higher powers possess a higher ability to separate the Ag colloids with sizes less than 40 nm. This is because more carbonaceous substances are generated at 600 mW, and these can then easily capture the large particles to form aggregate and precipitate over time, whereas the 300 mW LAL generates fewer carbon clusters, so that the size separation ability decreases, making it less efficient to separate the particles larger than 40 nm. The carbon ‘captors’ that originate from the LAL-induced decomposition of acetone have three forms, the carbon shells of Ag@C particles that capture small Ag particles to form truffle-like aggregates (Figure 4c and Figure 8c), the active wandering carbon clusters that gradually precipitate on particles to induce the formation of particle networks (Figure 8b,e), and the carbon nanosheets (Figure 9) that mainly capture the ultrasmall Ag particles with sizes less than 10 nm (Figure 9d–f). Recently, Escobar-Alarcón showed that the LAL of graphite in water could produce carbon nanosheets [52], in which they proposed that graphene exfoliation was the formation mechanism for carbon nanosheets. However, in our case, carbon comes from the decomposition of acetone molecules during LAL. Hence, the carbon nanosheets should form by the self-assembly of carbon clusters inside liquids, which can also explain the irregular structure shapes of nanosheets shown in Figure 9 and in Escobar-Alarcón et al. [52].

**Figure 10:**
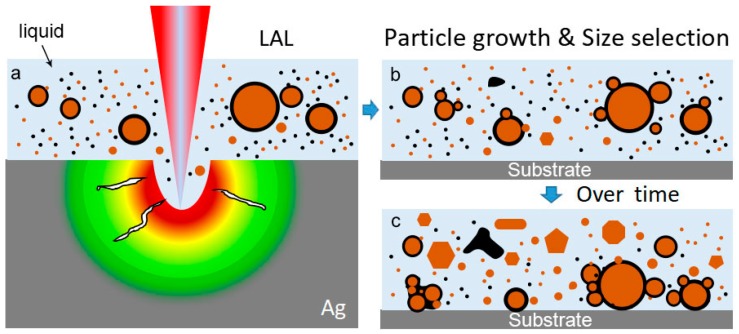
(**a**–**c**) Schematic of particle growth into polygonal nanocrystals, followed by the size separation of ultrasmall particles by large particle precipitation, due to carbon encapsulation. In the liquids, Ag and carbon are denoted by orange and black colored spheres, respectively.

Overall, the particle growth followed by the size separation phenomena was determined to occur during storage. The scenario is summarized in Figure 10. After long-period LAL, a large amount of Ag (orange colored spheres) and C (black colored spheres) clusters together to generate large Ag@C core-shell particles (Figure 10a). During the colloidal storage, the growth of ultrasmall surfactant-free Ag particles of 1–25 nm in diameter occurs through the Ostwald ripening mechanism (Figure 10b,c). Meanwhile, the soft carbon shells of large particles capture the surrounding small particles to form Ag@C-Ag aggregates. The aggregated particles precipitate at the bottom of the container (Figure 10b,c). The precipitation of the large particles cause the size distribution of the Ag particles in the supernatant to narrow by carbon encapsulation, which is termed size separation in this work. The self-size separation, with the aid of the large particle precipitation by carbon-encapsulation, can offset the disadvantage of the increased amount of large particles that are formed at a higher laser power, thus allowing a higher-productivity synthesis of metallic colloids with the uniform size distribution with that synthesized by the LAL at a lower laser power. The sediments may be technically separated by centrifugation or filtering to yield stable, monomodal Ag colloids in acetone.

### 2.2. Nanosecond Laser Ablation

**Figure 11:**
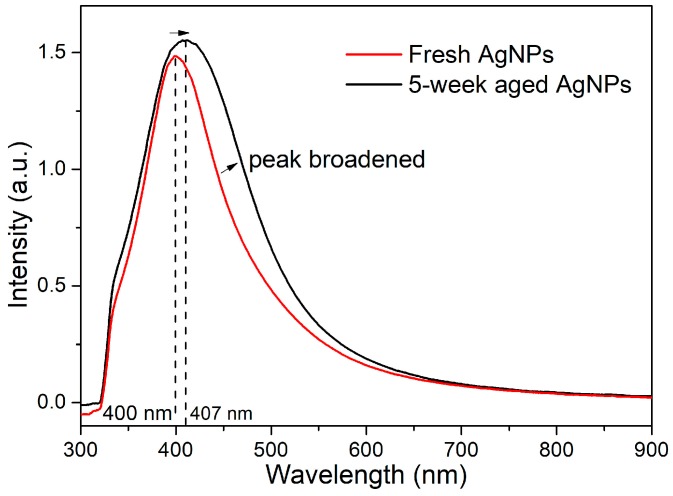
Absorption spectra for Ag NPs synthesized by ns laser ablation of Ag in acetone and then stored for five weeks.

**Figure 12:**
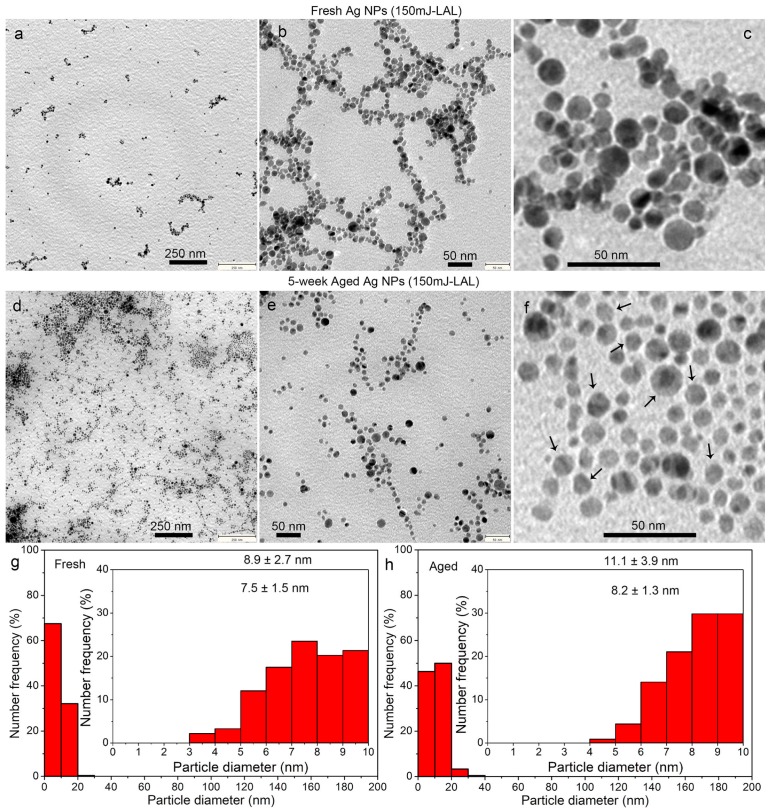
TEM images (**a**–**f**) and sizes distributions (**g**,**h**) of fresh (**a**–**c**,**g**) and five week (**d**–**f**,**h**) aged Ag particles synthesized by ns laser ablation of Ag in acetone at a pulse energy of 150 mJ.

Nanosecond laser ablation of Ag was performed in acetone for comparison, so as to investigate whether the spontaneous size separation and growth evolution into the polygonal crystals are specific for the fs-LAL, or whether they occur at a significantly longer pulse duration. Figure 11 shows the absorbance spectrum of the Ag NPs synthesized by the ns laser ablation of Ag in acetone, and that of the colloid after storage for five weeks. The SPR position of the Ag colloids was slightly redshifted from 400 nm to 407 nm after five weeks of storage, accompanied by the broadening of the SPR peak. Both indicate the change of the colloidal properties. Figure 12 shows the morphologies of the fresh and the aged Ag NPs, and their size distributions. Compared to the fresh Ag colloids with an average size of 8.93 ± 2.7 nm, the Ag NPs size was slightly increased to 11.1 ± 3.9 nm after storage for five weeks. Regarding the ultrasmall Ag NPs of 1–10 nm, the average size is increased from 7.5 ± 1.5 nm to 8.2 ± 1.3 nm after long-term storage. A significant decrease in the number ratio of the 1–10 nm particles from 68% to 47%, and the dramatic increase in the number ratio of the 10–20 nm particles from 22% to 50% (Figure 12g,h), provide evidence for the gradual growth of hte ns laser-synthesized Ag NPs in acetone during storage. The observation of more polygonal nanocrystals from the five week aged Ag colloid (Figure 12f) compared with the fresh colloid (Figure 12c) indicates that particle growth is always accompanied by the shape alteration of the metallic particles, regardless of the pulse duration used for colloid synthesis. Particle growth and simultaneous shape alternation are considered to constitute the main reason for the redshift and broadening of the SPR band (Figure 11).

However, compared to those particles synthesized by fs-LAL (Figure 2a–f and Figure 3a–f), the carbon shells were almost negligible for the ns-LAL generated Ag NPs (Figure 12a–f). No big particles larger than 40 nm were observed, which indicates that the fs-LAL, at high fluences (170 and 191 J/cm^2^ for 300 mW and 600 mW, respectively), causes more severe degradation of the acetone molecules than the ns-LAL at a low laser fluence (3 J/cm^2^). An FTIR study on the LAL in tetrahydrofuran showed that the carbonylic compounds were dominantly generated during ns-LAL, whereas the more olefinic species were dominantly generated during fs and picosecond (ps) laser ablation; therefore, the fs-LAL created more hydrophobic species [53]. If such hydrophobic species are also preferably formed during fs-LAL in acetone, then this could explain why their lower solubility triggers phase separation (into soft carbon shells, adsorbed on hydrophobic metal Ag), which supports the aggregation and sedimentation processes.

Regarding the polydispersity of the colloid, fs-LAL at high intensities leads to the formation of large Ag droplets jetting off the molten metal layer. This jetting is caused by Rayleigh instability, with the droplets evident even in front of an expanding cavitation bubble boundary [54]. These droplets solidify as large particles, so that the size distributions from the fs-LAL show stronger bimodality (even large spheres) compared to the ns-LAL.

Therefore, the ns-LAL in acetone yields colloids that are far less polydisperse than the fs-LAL. However, after the sedimentation or size separation of the aged fraction, the final colloid in the supernatant of the fs-LAL colloid has significantly smaller primary particles. Comparing the number ratios of 1–10 nm ultrasmall particles (80–90% for fs-LAL, Figure 2g and Figure 3g, vs. 68% for ns-LAL, Figure 12g), it can be easily deduced that fs-LAL is more efficient than ns-LAL for the generation of ultrasmall particles, at the expense of the mass yield lost during the size separation. In particular, the histograms from the TEM measurements show that the fs-LAL supernatant will contain a significantly larger portion of very small particles (<5 nm).

## 3. Materials and Methods

Silver colloids were synthesized by laser ablation of Ag in acetone by fs laser (FGPA μ Jewel D-1000-UG3, IMRA America Inc., Ann Arbor, MI, USA), with a pulse duration, wavelength, and repetition rate of 457 fs, 1045 nm, and 100 kHz, respectively. Two laser powers of 300 mW and 600 mW were adopted for the LAL. The spot sizes of the 300 mW LAL and 600 mW LAL were 15 and 20 μm, which gave the laser fluences were 170 of 191 J/cm^2^ for the 300 mW LAL and 600 mW LAL, respectively. A circular Ag plate with a diameter of 10 mm and a thickness of 1 mm was placed inside a glass dish filled with 8 mL acetone. The liquid thickness was ca. 5 mm above the target surface. The fs laser beam was then focused on the Ag plate surface using a 20× objective lens (NA = 0.45, Mitutoyo, Japan). An area of 3.5 × 3.5 mm^2^ was scanned using the parallel-line scanning method, described by the authors of [55,56,57], with an adjacent line interval of 5 μm. The scanning speed was set at 1 mm/s. Each ablation lasted 1 h to ensure long-period LAL [9].

The colloids that were just synthesized by the LAL, termed fresh colloids, were directly deposited onto the TEM grids (EMJapan, U1015, Tokyo, Japan, 20 nm thick carbon films on copper grids) and then characterized using TEM (Jeol, JEM-1230, Tokyo, Japan). UV-VIS spectroscopy (Shimadzu, UV-3600 Plus, Kyoto, Japan) was used to measure the absorption spectra during the colloidal aging every 30 min. More than 500 particles were measured by ImageJ to calculate the average particle sizes of the colloids. For the XRD (Rigaku, CuKα radiation (40 kV-30 mA), SmartLab-R 3kW, Tokyo, Japan) measurement, the colloids were centrifuged by a centrifuge (Eppendorf, Centrifuge 5430, Hamburg, Germany) at a rotation speed of 14,000 rpm for 10 min, and then deposited on a silicon wafer. The colloids were stored and sealed in glass containers at room temperature. After six months of storage, both the colloids in the liquids and the precipitated particles at the bottom of the glass container were characterized using TEM and UV-VIS spectroscopy. The surface chemistry of the Ag particles was analyzed using FTIR (Shimadzu, Prestige-21, Kyoto, Japan) and Raman spectrometers (LabRAM, He-Ne laser, 632 nm, 0.686 mW, Tokyo, Japan).

The nanosecond laser synthesis of the Ag colloids was performed using a Nd:YAG ns-laser (SpitLight DPSS250-100, InnoLas Laser GmbH, Krailling, Germany) at a pulse duration, wavelength, repetition rate, and pulse energy of 11 ns, 1064 nm, 100 Hz, and 150 mJ, respectively. The detected spot size on the target surface after ablation was 2.5 mm in diameter. The laser fluence is calculated to be 3 J/cm^2^. The Ag target was placed inside a glass cuvette filled with 3 mL of acetone. The liquid thickness above the target corresponded to the optical path (10 mm) of the glass cuvette used. The experiments were performed under continuous stirring of the liquid, with irradiation of the unfocused laser beam for 60 s. The colloids were characterized using UV-VIS spectroscopy (Thermo Scientific Evolution 201, Tokyo, Japan) and TEM (Zeiss, Type EM 910, Oberkochen, Germany), directly after synthesis and after five weeks of storage in sealed polypropylene tubes at room temperature.

## 4. Conclusions

The investigation of Ag colloids synthesized by the fs-LAL of Ag in acetone, at laser powers of 300 mW and 600 mW, confirmed the spontaneous growth of the ultrasmall particles and the spontaneous size separation during long-period storage. The FTIR spectroscopy analysis showed that the CO and CO_2_ molecules are adsorbed on the Ag particles to form Ag(CO)_n_ and Ag(CO_2_)_n_ complexes, which may contribute to the high stability of the supernatant Ag NPs. Carboxylate (COO–) and hydroxyl (OH–) species also conjugate on the Ag NPs, which may be the reason for the anisotropic growth of the Ag particles into the polygonal nanocrystals over time. Both the particle size increase and the shape transformation into the polygonal nanocrystals caused a redshift of the SPR bands from 404 nm to 414 nm. The carbonaceous species generated from the acetone decomposition and pyrolysis during the LAL gradually adsorbed onto the large particles, and the soft carbon shells of the Ag@C particles also captured smaller particles, to form Ag@C-Ag aggregates. Particle aggregation and the formation of Ag–C networks significantly compromise the stability of the large Ag particles and cause their gradual precipitation during long-period storage, which leaves ultrasmall particles behind in the liquid supernatant. A higher size separation ability is endowed with the colloids obtained by fs-LAL at a higher power (600 mW), which possibly benefits from a larger amount of carbon captors, because of the stronger decomposition or pyrolysis of the solvent, which possibly creates olefinic species with a low solubility in acetone. Such phase-separating, carbon-induced size separation induced by the colloids themselves may be helpful to conquer the challenging issue of the wide (i.e., bimodal) size distribution of the metallic particles generated by ultrashort-pulsed-LAL. When the growth is terminated and mechanically fractionated from the supernatant as a precipitate, the resulting supernatant of the fs-LAL-derived colloids bears a significantly higher fraction of very small particles (≤5 nm), compared to the ns-LAL. On the other hand, the ns-LAL of the Ag in acetone yields a monomodal particle size distribution with lower polydispersity, and the particle size also grows during storage, but without size focusing via precipitation, because no thick organic shells were observed by the TEM observation.

The particle growth is often neglected for the LAL-generated metallic particles. This must be taken into account when characterizing the particle state by TEM and before using these particles for practical applications. If the characterization and application were conducted at different times and particle growth had occurred, then the mismatch of the particle properties and their performances, particularly for size-sensitive applications such as catalysis and biology, would mislead the interpretation of all of the experimental results.

## References

[1] Zhang D., Gökce B., Barcikowski S. (2017). Laser synthesis and processing of colloids: Fundamentals and applications. Chem. Rev..

[2] Zeng H., Du X.W., Singh S.C., Kulinich S.A., Yang S., He J., Cai W. (2012). Nanomaterials via laser ablation/irradiation in liquid: A review. Adv. Funct. Mater..

[3] Xiao J., Liu P., Wang C.X., Yang G.W. (2017). External field-assisted laser ablation in liquid: An efficient strategy for nanocrystal synthesis and nanostructure assembly. Prog. Mater. Sci..

[4] Zhang J., Claverie J., Chaker M., Ma D. (2017). Colloidal metal nanoparticles prepared by laser ablation and their applications. ChemPhysChem.

[5] Zhang J., Chen G., Chaker M., Rosei F., Ma D. (2013). Gold nanoparticle decorated ceria nanotubes with significantly high catalytic activity for the reduction of nitrophenol and mechanism study. Appl. Catal. B.

[6] Hebié S., Holade Y., Maximova K., Sentis M., Delaporte P., Kokoh K.B., Napporn T.W., Kabashin A.V. (2015). Advanced electrocatalysts on the basis of bare au nanomaterials for biofuel cell applications. ACS Catal..

[7] Zhang D., Liu J., Li P., Tian Z., Liang C. (2017). Recent advances in surfactant-free, surface charged and defect-rich catalysts developed by laser ablation and processing in liquids. ChemNanoMat.

[8] Zhang J., Chaker M., Ma D. (2017). Pulsed laser ablation based synthesis of colloidal metal nanoparticles for catalytic applications. J. Colloid Interface Sci..

[9] Zhang D., Liu J., Liang C. (2017). Perspective on how laser-ablated particles grow in liquids. Sci. China Phys. Mech. Astron..

[10] Jendrzej S., Gökce B., Amendola V., Barcikowski S. (2016). Barrierless growth of precursor-free, ultrafast laser-fragmented noble metal nanoparticles by colloidal atom clusters—A kinetic in situ study. J. Colloid Interface Sci..

[11] Poletti A., Fracasso G., Conti G., Pilot R., Amendola V. (2015). Laser generated gold nanocorals with broadband plasmon absorption for photothermal applications. Nanoscale.

[12] Zhou L., Zhang H., Bao H., Liu G., Li Y., Cai W. (2017). Onion-structured spherical mos_2_ nanoparticles induced by laser ablation in water and liquid droplets’ radial solidification/oriented growth mechanism. J. Phys. Chem. C.

[13] Kabashin A.V., Meunier M. (2003). Synthesis of colloidal nanoparticles during femtosecond laser ablation of gold in water. J. Appl. Phys..

[14] Rehbock C., Merk V., Gamrad L., Streubel R., Barcikowski S. (2013). Size control of laser-fabricated surfactant-free gold nanoparticles with highly diluted electrolytes and their subsequent bioconjugation. Phys. Chem. Chem. Phys..

[15] Liu J., Liang C., Tian Z., Zhang S., Shao G. (2013). Spontaneous growth and chemical reduction ability of Ge nanoparticles. Sci. Rep..

[16] Zhang D., Lau M., Lu S., Barcikowski S., Gökce B. (2017). Germanium sub-microspheres synthesized by picosecond pulsed laser melting in liquids: Educt size effects. Sci. Rep..

[17] Zhang D., Gökce B., Notthoff C., Barcikowski S. (2015). Layered seed-growth of agge football-like microspheres via precursor-free picosecond laser synthesis in water. Sci. Rep..

[18] Zhang D., Ma Z., Spasova M., Yelsukova A.E., Lu S., Farle M., Wiedwald U., Gökce B. (2017). Formation mechanism of laser-synthesized iron-manganese alloy nanoparticles, manganese oxide nanosheets and nanofibers. Part. Part. Syst. Charact..

[19] Liang C., Sasaki T., Shimizu Y., Koshizaki N. (2004). Pulsed-laser ablation of mg in liquids: Surfactant-directing nanoparticle assembly for magnesium hydroxide nanostructures. Chem. Phys. Lett..

[20] Zhang H., Duan G., Li Y., Xu X., Dai Z., Cai W. (2012). Leaf-like tungsten oxide nanoplatelets induced by laser ablation in liquid and subsequent aging. Cryst. Growth Des..

[21] Niu K.Y., Yang J., Kulinich S.A., Sun J., Li H., Du X.W. (2010). Morphology control of nanostructures via surface reaction of metal nanodroplets. J. Am. Chem. Soc..

[22] Schaumberg C.A., Wollgarten M., Rademann K. (2014). Metallic copper colloids by reductive laser ablation of non metallic copper precursor suspensions. J. Phys. Chem. A.

[23] Liu J., Liang C., Zhu X., Lin Y., Zhang H., Wu S. (2016). Understanding the solvent molecules induced spontaneous growth of uncapped tellurium nanoparticles. Sci. Rep..

[24] Wu C.-H., Chen S.-Y., Shen P. (2014). Special grain boundaries of anatase nanocondensates by oriented attachment. CrystEngComm.

[25] Wang H., Odawara O., Wada H. (2016). Facile and chemically pure preparation of YVO_4_: Eu^3+^ colloid with novel nanostructure via laser ablation in water. Sci. Rep..

[26] Huang C.-C., Yeh C.-S., Ho C.-J. (2004). Laser ablation synthesis of spindle-like gallium oxide hydroxide nanoparticles with the presence of cationic cetyltrimethylammonium bromide. J. Phys. Chem. B.

[27] Xia Y., Xiong Y., Lim B., Skrabalak S.E. (2009). Shape-controlled synthesis of metal nanocrystals: Simple chemistry meets complex physics?. Angew. Chem. Int. Ed..

[28] Tsuji T., Kakita T., Tsuji M. (2003). Preparation of nano-size particles of silver with femtosecond laser ablation in water. Appl. Surf. Sci..

[29] Pyatenko A., Shimokawa K., Yamaguchi M., Nishimura O., Suzuki M. (2004). Synthesis of silver nanoparticles by laser ablation in pure water. Appl. Phys. A.

[30] Streubel R., Bendt G., Gökce B. (2016). Pilot-scale synthesis of metal nanoparticles by high-speed pulsed laser ablation in liquids. Nanotechnology.

[31] Tilaki R.M., Mahdavi S.M. (2006). Stability, size and optical properties of silver nanoparticles prepared by laser ablation in different carrier media. Appl. Phys. A.

[32] Tsuji T., Tsuji M., Hashimoto S. (2011). Utilization of laser ablation in aqueous solution for observation of photoinduced shape conversion of silver nanoparticles in citrate solutions. J. Photochem. Photobiol. A.

[33] Tsuji T., Mizuki T., Ozono S., Tsuji M. (2009). Laser-induced silver nanocrystal formation in polyvinylpyrrolidone solutions. J. Photochem. Photobiol. A.

[34] Tsuji T., Higuchi T., Tsuji M. (2005). Laser-induced structural conversions of silver nanoparticles in pure water—Influence of laser intensity. Chem. Lett..

[35] Tsuji T., Kikuchi M., Kagawa T., Adachi H., Tsuji M. (2017). Morphological changes from spherical silver nanoparticles to cubes after laser irradiation in acetone–water solutions via spontaneous atom transportation process. Colloids Surf. A.

[36] Tsuji T., Nakanishi M., Mizuki T., Ozono S., Tsuji M., Tsuboi Y. (2012). Preparation and shape-modification of silver colloids by laser ablation in liquids: A brief review. Sci. Adv. Mater..

[37] Kőrösi L., Rodio M., Dömötör D., Kovács T., Papp S., Diaspro A., Intartaglia R., Beke S. (2016). Ultra-small, ligand-free Ag nanoparticles with high antibacterial activity prepared by pulsed laser ablation in liquid. J. Chem..

[38] Tiedemann D., Taylor U., Rehbock C., Jakobi J., Klein S., Kues W.A., Barcikowski S., Rath D. (2014). Reprotoxicity of gold, silver, and gold–silver alloy nanoparticles on mammalian gametes. Analyst.

[39] Robertson J. (2002). Diamond-like amorphous carbon. Mater. Sci. Eng..

[40] Sengan M., Veeramuthu D., Veerappan A. (2018). Photosynthesis of silver nanoparticles using durio zibethinus aqueous extract and its application in catalytic reduction of nitroaromatics, degradation of hazardous dyes and selective colorimetric sensing of mercury ions. Mater. Res. Bull..

[41] Bastús N.G., Merkoçi F., Piella J., Puntes V. (2014). Synthesis of highly monodisperse citrate-stabilized silver nanoparticles of up to 200 nm: Kinetic control and catalytic properties. Chem. Mater..

[42] An J., Tang B., Ning X., Zhou J., Xu S., Zhao B., Xu W., Corredor C., Lombardi J.R. (2007). Photoinduced shape evolution: From triangular to hexagonal silver nanoplates. J. Phys. Chem. C.

[43] Yu B., Shi Y., Yuan B., Qiu S., Xing W., Hu W., Song L., Lo S., Hu Y. (2015). Enhanced thermal and flame retardant properties of flame-retardant-wrapped graphene/epoxy resin nanocomposites. J. Mater. Chem. A.

[44] Mansur H.S., Sadahira C.M., Souza A.N., Mansur A.A.P. (2008). Ftir spectroscopy characterization of poly (vinyl alcohol) hydrogel with different hydrolysis degree and chemically crosslinked with glutaraldehyde. Mater. Sci. Eng. C.

[45] Yajima T., Uchida H., Watanabe M. (2004). In-situ ATR-FTIR spectroscopic study of electro-oxidation of methanol and adsorbed CO at Pt–Ru alloy. J. Phys. Chem. B.

[46] Pritchard J., Catterick T., Gupta R.K. (1975). Infrared spectroscopy of chemisorbed carbon monoxide on copper. Surf. Sci..

[47] Dong C., Wirasaputra A., Luo Q., Liu S., Yuan Y., Zhao J., Fu Y. (2016). Intrinsic flame-retardant and thermally stable epoxy endowed by a highly efficient, multifunctional curing agent. Materials.

[48] Liang B., Zhou M., Andrews L. (2000). Reactions of laser-ablated Ni, Pd, and Pt atoms with carbon monoxide:  Matrix infrared spectra and density functional calculations on M(CO)_n_ (n = 1–4), M(CO)_n_^−^ (n = 1–3), and M(CO)_n_^+^ (n = 1–2), (M = Ni, Pd, Pt). J. Phys. Chem. A.

[49] Ramis G., Busca G., Lorenzelli V. (1991). Low-temperature CO_2_ adsorption on metal oxides: Spectroscopic characterization of some weakly adsorbed species. Mater. Chem. Phys..

[50] Liang B., Andrews L. (2000). Reactions of laser-ablated Ag and Au atoms with carbon monoxide: Matrix infrared spectra and density functional calculations on Ag(CO)_n_ (n = 2, 3), Au(CO)_n_ (n = 1, 2) and M(CO)_n_^+^ (n = 1–4; M = Ag, Au). J. Phys. Chem. A.

[51] Mikhlin Y.L., Vorobyev S.A., Saikova S.V., Vishnyakova E.A., Romanchenko A.S., Zharkov S.M., Larichev Y.V. (2018). On the nature of citrate-derived surface species on Ag nanoparticles: Insights from X-ray photoelectron spectroscopy. Appl. Surf. Sci..

[52] Escobar-Alarcón L., Espinosa-Pesqueira M.E., Solis-Casados D.A., Gonzalo J., Solis J., Martinez-Orts M., Haro-Poniatowski E. (2018). Two-dimensional carbon nanostructures obtained by laser ablation in liquid: Effect of an ultrasonic field. Appl. Phys. A.

[53] Van’t Zand D.D., Nachev P., Rosenfeld R., Wagener P., Pich A., Klee D., Barcikowski S. (2012). Nanocomposite fibre fabrication via in situ monomer grafting and bonding on laser-generated nanoparticles. J. Laser Micro/Nanoeng..

[54] Shih C.-Y., Streubel R., Heberle J., Letzel A., Shugaev M., Wu C., Schmidt M., Gokce B., Barcikowski S., Zhigilei L. (2018). Two mechanisms of nanoparticle generation in picosecond laser ablation in liquids: The origin of the bimodal size distribution. Nanoscale.

[55] Zhang D., Chen F., Fang G., Yang Q., Xie D., Qiao G., Li W., Si J., Hou X. (2010). Wetting characteristics on hierarchical structures patterned by a femtosecond laser. J. Micromech. Microeng..

[56] Zhang D., Chen F., Yang Q., Si J., Hou X. (2011). Mutual wetting transition between isotropic and anisotropic on directional structures fabricated by femotosecond laser. Soft Matter.

[57] Zhang D., Chen F., Yang Q., Yong J., Bian H., Ou Y., Si J., Meng X., Hou X. (2012). A simple way to achieve pattern-dependent tunable adhesion in superhydrophobic surfaces by a femtosecond laser. ACS Appl. Mater. Interfaces.

